# Null and Void? Errors in Meta-analysis on Perceptual Disfluency and Recommendations to Improve Meta-analytical Reproducibility

**DOI:** 10.1007/s10648-020-09579-1

**Published:** 2021-02-03

**Authors:** Sophia C. Weissgerber, Matthias Brunmair, Ralf Rummer

**Affiliations:** 1 Institute of Psychology, Department of Cognitive Psychology, University of Kassel, Holländische Str. 36-38, 34127 Kassel, Germany; 2 University of Würzburg, Röntgenring 10, 97070 Würzburg, Germany

**Keywords:** Disfluency effect, Transparency, Meta-analytical standards, Open-science, Reproducibility

## Abstract

In the 2018 meta-analysis of *Educational Psychology Review* entitled “Null effects of perceptual disfluency on learning outcomes in a text-based educational context” by Xie, Zhou, and Liu, we identify some errors and inconsistencies in both the methodological approach and the reported results regarding coding and effect sizes. While from a technical point of view the meta-analysis aligns with current meta-analytical guidelines (e.g., PRISMA) and conforms to general meta-analytical requirements (e.g., considering publication bias), it exemplifies certain insufficient practices in the creation and review of meta-analysis. We criticize the lack of transparency and negligence of open-science practices in the generation and reporting of results, which complicate evaluation of the meta-analytical reproducibility, especially given the flexibility in subjective choices regarding the analytical approach and the flexibility in creating the database. Here we present a framework applicable to pre- and post-publication review on improving the *Methods Reproducibility* of meta-analysis. Based on considerations of the transparency and openness (TOP)-guidlines (Nosek et al. Science 348: 1422–1425, 2015), the Reproducibility Enhancement Principles (REP; Stodden et al. Science 354:1240–1241, 2016), and recommendations by Lakens et al. (BMC Psychology 4: Article 24, 2016), we outline *Computational Reproducibility* (Level 1), *Computational Verification* (Level 2), *Analysis Reproducibility* (Level 3), and *Outcome Reproducibility* (Level 4). Applying reproducibility checks to TRANSFER performance as the chosen outcome variable, we found Xie’s and colleagues’ results to be (rather) robust. Yet, regarding RECALL performance and the moderator analysis, the identified problems raise doubts about the credibility of the reported results.

## Introduction

Recent years have seen an accumulation of unprecedented heterogeneous findings on perceptual disfluency in learning (i.e., harder-to-read text improves memory; *positive disfluency effect*: e.g., Weltman and Eakin 2014; Weissgerber and Reinhard 2017; *null-effect*: e.g., Eitel and Kuehl 2016; Rummer et al. 2016; *negative effect*: e.g., Kuehl et al. 2014a; Pieger et al. 2017) paired with heated debates on potential reasons and moderators (e.g., Kuehl et al. 2014b; Kuehl and Eitel 2016; Oppenheimer and Alter 2014). Both the heterogeneity of the findings and the search for potential moderators have called for a meta-analysis of studies investigating the influence of perceptual disfluency on learning. In 2018, such a meta-analysis was published in this journal titled “Null effects of perceptual disfluency on learning outcomes in a text-based educational context” (Xie et al. 2018). As the title of the meta-analysis indicates, the pooled effect size for disfluency on RECALL and TRANSFER performance was almost null.

Such a meta-analytically derived result has a particularly high impact because a meta-analysis summarizes multiple primary studies and is thus considered more trustworthy than single studies. The credibility of meta-analyses rests, though, on their underlying quality. Several guidelines (e.g., MARS: APA 2008; PRISMA guidelines: Moher et al. 2009) layout quality criteria, and Xie’s and colleagues’ meta-analysis positively conforms to these standards. Xie and colleagues also took care to include results from the gray literature, such as dissertations, conference presentations, and unpublished research, and they displayed a funnel-plot to identify potential bias in the included published primary studies.

Producing a credible and impactful meta-analysis relies, however, on not only following quality standards in guidelines and attending to publication bias; it also depends on *careful, transparent*, *comprehensible*, and *reproducible* methodological conduct. In this respect, we noticed several shortcomings in Xie’s and colleagues’ meta-analysis. We acknowledge that transparency and reproducibility issues in general also plague other meta-analyses and have proven to be more (e.g., Gøtzsche et al. 2007) or less consequential (e.g., Jones et al. 2005; Maassen et al. 2020). We further acknowledge that following meta-analytical guidelines closely improves meta-analytical quality (e.g., Schalken and Rietbergen 2017). Nevertheless, adhering to the quality standards of general guidelines on generating and reporting meta-analyses alone seems insufficient, as Xie’s and colleagues’ meta-analysis indicates. Guidelines are no guarantee for authors’ reproducible conduct (Lakens et al. 2016) and no guarantee that another researcher will invest the time and resources trying to reproduce the reported results. They are also no guarantees that a careful reviewer will meticulously check a meta-analysis regarding reproducibility and transparency aspects.

We present the current paper as an opportunity to build on previous recommendations (Lakens et al. 2016; Stodden et al. 2016) and the transparency and openness (TOP-) guidelines (Nosek et al. 2015; see also https://osf.io/9f6gx/) to suggest further improvements for current practices, applicable to both *pre-* and *post*-review of manuscripts. We present a *framework* targeted at facilitating the reproducibility of meta-analysis. In a nutshell, we address numerical accuracy (*Computational Reproducibility*: Level 1) and numerical validity (*Computational Verification*: Level 2). We particularly attend to variations due to flexibility regarding the analytical approach (*Analysis Reproducibility*: Level 3) and variations due to the flexibility in creating the database (*Outcome Reproducibility*: Level 4). As such, the framework introduces a systematic approach to address how others (including reviewers) can check and “re-analyze the data to examine how sensitive the results are to subjective choices” (Lakens et al. 2016; page 4), but we extend our consideration to include subjective choices in the dataset creation.^1^ The appendices and supplemental materials act as a demonstration of the approach regarding TRANSFER performance of Xie et al. (2018). While the framework is applicable to the review of meta-analysis post-publication, we advocate for the application as a tool for quality control *prior* to publication.

## Guidelines and Inconsistencies in Xie’s et al.’s (2018) Meta-analysis

At first glance, the meta-analysis of Xie and colleagues appears well done and in adherence with widely accepted guidelines on meta-analytical standards (MARS-guidelines: APA 2008; PRISMA guidelines: Moher et al. 2009; PRISMA-P guidelines: Moher et al. 2015). For example, Xie and colleagues report on most points outlined by both PRISMA recommendations, such as reporting on their literature search strategy, eligible criteria for inclusion, and coding of studies (related to the moderator variables), and Xie and colleagues give an overview of their rationale for effect size calculation and overall analysis approach including correction for biases. In Appendix L1: Workflow and PRISMA guidelines of Xie.pdf (https://osf.io/5v8m2/), we present a short summary of information reported by Xie et al. 2018, and a comparison with both PRISMA guidelines (PRISMA guidelines: Moher et al. 2009; PRISMA-P guidelines: Moher et al. 2015).

Xie’s and colleagues’ approach conforms to the quality standards set in these recommendations with occasionally minor deviations or omissions of information: For example, instead of presenting “the full electronic search strategy for at least one database, including any limits used, such that it could be repeated” (PRISMA 2009: #8; cf. PRISMA-P 2015:#10), Xie et al. (2018) provide the used keywords and searched databases. Instead of a flow diagram with numbers of studies screened, assessed for eligibility, and included in the review with reasons for exclusions at each stage (PRISMA 2009: #17), Xie and colleagues summarized most of the eligibility information in text form (except for exclusions). An apparent deviation from these guidelines that we noticed was not providing information on how many coders extracted the effect sizes of the primary studies and what their intercoder reliability was (PRISMA, 2009: #10; cf. PRISMA-P, 2015: #11c). We thus looked more carefully into coding and effect sizes, given that coding decisions and effect sizes can be a critical source of error and inconsistencies for meta-analytical reproducibility (e.g., Lakens et al. 2016; Maassen et al. 2020).

First, we discovered some *objective* coding errors concerning the report of our own experiments (Rummer et al. 2016; Weissgerber and Reinhard 2017). Rummer et al. (2016) ran three experiments as close replications of the original study by Diemand-Yauman et al. (2011; Experiment 1). None of their three experiments replicated the disfluency effect. In Figure 2 of Xie’s and colleagues’ meta-analysis, however, Experiment 2 by Rummer et al. (2016) was misreported as favoring the disfluency effect. In fact, Rummer's et al.’s (2016) results are rather indicative of the opposite: a small advantage of fluency. In contrast, Weissgerber and Reinhard (2017) ran an experiment manipulating perceptual disfluency including two performance tests, immediately after learning and two weeks later. While a null effect of font manipulation was found immediately, two weeks later a clear performance advantage for disfluent fonts was observed. In Figure 2 of the meta-analysis, Weissgerber's and Reinhard’s (2017) delayed condition was misreported as evidence against the disfluency effect, when in fact the results are evidence in favor of disfluency. Hence, both studies (Rummer et al. 2016; Weissgerber and Reinhard 2017) were misrepresented and incorrectly incorporated into the analysis.

Based on these two unsystematic and perchance observations, we decided to check and reproduce more systematically a broader sample of studies referenced in the meta-analysis. We strategically chose to check first the papers authored by Eitel and/or Kuehl, given their active contribution to the disfluency literature (e.g., Eitel et al. 2014; Kuehl et al. 2014a) and debate (e.g., Kuehl et al. 2014b; Kuehl and Eitel 2016). We chose to check RECALL performance first, given that RECALL as outcome is rather clear (i.e., what constitutes RECALL opposed to what constitutes TRANSFER). For effect size recalculation, we followed the workflow of Maassen et al. (2020; their Figure 1, page 5): We identified appropriate (composite) effects in primary studies based on available information in the meta-analysis; when more than one (composite) effect calculation was possible, we calculated multiple logically possible effects and chose the effect size closest to the primary effect reported in the meta-analysis.

As a basis for our recalculations of primary effect sizes, we already had extracted and reviewed all available information of Xie’s and colleagues’ (2018) meta-analysis for the comparisons to the PRISMA guidelines (Moher et al. 2009, 2015; see Appendix L1, https://osf.io/5v8m2/). Based on the reporting standards of the PRISMA guidelines, there is no basis for sharp criticism of Xie’s et al.’s (2018) work. In line with the guidelines on data items and outcomes (PRISMA 2009: #11; cf. PRISMA-P 2015: #12 and #13), Xie and colleagues listed the variables for which data were extracted, including some general assumptions and simplifications made for coding decisions (see Xie’s et al.’s page 752 and following pages).^2^ In terms of calculation of effect sizes and analysis, Xie and colleagues conform to the required information on stating the summary measures (PRISMA 2009: #13) and on describing the *general* method for handling data and combining results, including criteria for meta-analytical synthesis (PRISMA 2009: #14; cf. PRISMA-P 2015: #15a and #15b). Xie et al. (2018) present study characteristics for which data were extracted for each study (PRISMA 2009: #18; cf. PRISMA-P 2015: #15a and #15b; e.g., see Table 1 of Xie et al. 2018), apart from providing the exact underlying text source. In their Figure 2, Xie et al. (2018) present the results of the individual studies as simple summary data, including effect estimates and confidence intervals with a forest plot for the calculated effect size estimate (but not for each underlying intervention group chosen and their analytical processing; PRISMA 2009: #20).

**Table 1:** Overview of Modular Aspects of Methods Reproducibility to Improve Meta-analysis

Methods reproducibility^b^	Goal	Original author(s) provide:	Independent researcher(s), e.g., designated and responsible co-author, or reviewer, or journal associate	Example / files
**Level 1** (prior publication)	**Computational Reproducibility** (Are the results numerically the same when reproduced?)	- Original data (including raw data used to estimate effect sizes and further variables) - Original analysis code (understandable) - Description of used analysis tools (software and version, etc.)	• Tests originally provided materials for completeness, usability, and understandability. • Compares based on exactly these materials the numbers reported in the meta-analysis and the numbers produced by the analysis output. • Criteria: reproduction percentage, correct 95%.	Note: Since no data or code files were provided by Xie and colleagues, we could not apply Level 1 checks as outlined, but instead adopted a variant (e.g., compiled a document and applied PRISMA-checks and reproduced a selection of meta-analytical results). **Main**: see **Appendix L1**: Workflow and PRISMAguidelines of Xie.pdf, https://osf.io/5v8m2/ **See Supplemental L1**: RECALL SMD of Eitel and Kuehl (2016) Kuehl et al. (2014a) Eitel et al. (2014).xlsx, https://osf.io/r6d5e/
**Level 2** (prior publication)	**Computational Verification** (Are the provided information and resources, like analysis code, error-free, clear, and accurate to allow meaningful reproduction?)	- Original data (including raw data used to estimate effect sizes and further variables) - Original analysis code (understandable) - Description of used analysis tools (software and version, etc.) - *Codebook (well-curated)* - *Computational formulas (especially for dealing with different design variants)* - *Original workflow (including all processing steps)* - *Log of subjective decisions (*e.g.*, study (sub-)groups, effect size selection, and coding decisions)* - *Eligible criteria for study selection (and if possible, list of and reason for excluded papers)* - *Additionally obtained information (*e.g.*, from authors of primary studies)*	• Searches data and analysis code and workflow for minor errors, mistakes, and any uncertainties ensuring accurate data and analysis code. • Reflects on and verifies the accuracy of the general analysis approach and specific analysis steps taken by original authors. • Checks four sources of error (cf. Lakens et al. 2016) • Erroneous calculation of effect sizes • Inconsistent calculation of effect sizes across studies • Incorrect inclusion of effect sizes • Incorrect calculation of meta-analytic effect size	Note: Similar to Level 1, we could not conduct Level 2 checks as outlined, but we adopted a variant of Level 2 checks. **Main:** see **Appendix L2**: Check of dataset, effect sizes, and workflow of Xie.pdf, https://osf.io/7gj23/ **Supplemental L2-A**: OVERVIEW check coding decisions Table 1 of Xie (TRANSFER).xlsx, https://osf.io/49cuk/ **Supplemental L2-B**: INFO check coding decisions of Xie (TRANSFER).docx, https://osf.io/5t9jn/ **Underlying data/analysis files:** L2_Computational Verification.cma, https://osf.io/kzb95/ L2_Eitel and Kuehl (2016).cma, https://osf.io/maz8d/ L2_Faber et al. (2017).cma, https://osf.io/ucvqa/ L2_Eitel et al. (2014) coding error Exp 3.cma, https://osf.io/9jb6e/ L2_Eitel et al. (2014) corrected.cma, https://osf.io/g2wu9/ L2_Kuehl et al. (2014a).cma, https://osf.io/exks3/ L2_Lehmann et al. (2016).cma, https://osf.io/zwn9r/ L2_Seufert et al. (2017).cma, https://osf.io/pcg3b/ L2_Whitehouse (2011)_all.cma, https://osf.io/f3654/ L2_Weltman and Eakin (2014).cma, https://osf.io/wkd4y/ L2_Whitehouse (2011) pooling.xlsx, https://osf.io/rqp8e/
**Level 3** (prior alongside. or post publication)	**Analysis Reproducibility** (Are the conducted analyses robust and reproducible in light of researchers’ flexibility in analytical choices?)		• Applies multiverse and specification-curve approach to the meta-analysis (see Voracek et al. 2019).^a^ • Compares effect size variations depending on various (eligible) analysis approaches and analysis specifications with effect size(s) obtained by the original authors of the meta-analysis. • Or at least, applies own analytical tool and own analytical approaches *based on a sub-sample of the provided original data and (a) randomly or strategically chosen dependent variable(s)* of the originally reported results. • Checks if similar results as those of the original authors can be reproduced when fitting similar models (not the same models, see Level 1). • Identifies multiple different but defensible (and most plausible) analysis approaches/models and checks if similar results as those of the original authors can be reproduced. • Deposits beforehand a reproducibility plan or preregistered protocol including evaluation criteria and metrics to avoid bias.	Level 3 checks are performed as outlined for TRANSFER as chosen outcome variable on the (corrected) original dataset; illustrated are own similar and DISsimilar analysis (given there already is a worked example on specification curve for meta-analysis, Voracek et al. 2019). **Main:** see **Appendix L3**: Similar and DISsimilar analysis approach.pdf, https://osf.io/zdf8v/ See **Supplemental L3-A**: Our subjective decision tree (multiverse pool).pdf, https://osf.io/2k86t/ See **Supplemental L3-B**: Our workflow (subjective decisions).pdf, https://osf.io/eyvdp/ **Underlying data/analysis files:** L3_00S_similar analysis (all).cma, https://osf.io/hgr8j/ L3_01D_DISsimilar analysis (all with moderator covariate inclusion).cma, https://osf.io/wc42g/ L3_02D_DISsimilar analysis (without Whitehouse 2011).cma, https://osf.io/baqfp/ L3_03D_DISsimilar analysis (without Whitehouse 2011 & Kuehl et al. 2014a).cma, https://osf.io/ng5q6/
**Level 4** (prior alongside. or post publication)	**Outcome Reproducibility** (Are the reported outcomes reproducible despite variability due to subjective decisions related to the creation of the database and researchers’ flexibility in analytical choices?)		• Applies multiverse and specification-curve approach or at least applies own analytical tool and own analytical approaches *based on own (re-)creation of (a small portion of) the database by own coding of primary studies*. • Checks original and *recreated* data set for coding mistakes and inappropriate choices (e.g., with (sub-)group selection or inconsistent use of specification within and between primary studies). • Includes alternative subjective data coding decisions in *recreated data set* to compute alternative results. • Compares if and how much the results deviate while applying the same analytical tools and approach as used in Level 1. • Compares if and how much the results deviate while applying the analytical choices outlined in Level 3. • Deposits beforehand a reproducibility plan or preregistered protocol including evaluation criteria and metrics to avoid bias.	Level 4 checks are performed as outlined for TRANSFER as chosen outcome variable and based on own creation of the database by own coding of all respective primary studies. **Main:** see **Appendix L4**: Similar and DISsimilar analysis approach.pdf, https://osf.io/jwng6/ **Underlying data/analysis files:** L4_00S_similar analysis (all).cma, https://osf.io/pkjyd/ L4_01D_DISsimilar analysis (all with moderator covariate inclusion).cma, https://osf.io/re9bx/ L4_02D_DISsimilar analysis (without Whitehouse 2011).cma, https://osf.io/8xvb2/ L4_03D_DISsimilar analysis (without Whitehouse 2011 & Kuehl et al. 2014a).cma, https://osf.io/36qs2/

^a^Of course, this only makes sense until a multiverse and specification-curve approach for meta-analysis becomes standard procedure. Otherwise the verification of the specification curve results is required (= Level 1 and Level 2; skipping Level 3 and proceeding to Level 4).

^b^Note: Goodman’s and colleagues’ Goodman et al. (2016) definition of Methods Reproducibility emphasizes precision regarding the implementation of the computational procedures “as exactly as possible, with the same data and tools, to obtain the same results.” (Goodman et al. 2016, page 2). This is reflected in our Level 1 conceptualization of Methods Reproducibility as Computational Reproducibility. Our conception of Level 3’s Analytical Reproducibility builds on but necessarily removes this precision constraint by considering researcher’s flexibility in analytical choices (and tools). Likewise, our conceptualization of Level 4 further removes the constraint of using *exactly* the same data source. The logic of the four levels is to move from reliability to include validity aspects.

We must state that it was difficult and time-consuming to reconstruct Xie’s and colleagues’ exact approach. Their methodological description (and therefore our reproduction attempt) is characterized by many ambiguous effect size computations, despite the fact that Xie and colleagues largely followed PRISMA guidelines (Moher et al. 2009, 2015). Such problems are not confined to Xie’s and colleagues’ meta-analysis (cf. Maassen et al. 2020); nevertheless, they undermine reproducibility, given that it was often unclear to us exactly which effect sizes were selected, whether and how they were combined, and exactly which calculations steps and formula were applied.

The PRISMA guidelines (Moher et al. 2009, 2015) on reporting the effect size selection and computations are more generic and do not require a *precise* documentation at the level of each study or each calculated effect size including underlying subjective decisions. The PRISMA guidelines require an overall description and summary, which only in some exceptions refers directly to specific individual effect sizes. Even if more stringent adherence to the PRISMA guidelines increases the meta-analytical reproducibility (cf. Schalken and Rietbergen 2017), it does not automatically follow that the meta-analytical results are reproducible; this applies in particular to effect sizes, especially if no specific information for each study and each effect size and no dataset or other resources were made available (as required by newer meta-analytical guidelines, e.g., Lakens et al. 2016).

We followed the description of Xie’s et al.’s approach (as much as we could reconstruct it) and used the information in their Figure 2 to estimate whether and how (sub-)groups in a study were combined. Accordingly, we recalculated the standardized mean difference (SMD^3^) of RECALL for Eitel and Kuehl (2016) based on Xie’s et al.’s information on “Calculation of Effect Sizes and Analysis” (see page 754^4^ of Xie et al. 2018). Xie’s and colleagues’ Figure 2 indicated that Xie et al. (2018) adopted separate calculations for each (sub-)group of Eitel and Kuehl (2016; i.e., calculation of SMD based on Cohen’s d for low-test expectancy / high test expectancy). Based on the original means and standard errors reported in Eitel and Kuehl (2016, their Table 1), we obtained the same values for Cohen’s d as those presented by Xie and colleagues in their Figure 2 for RECALL. Yet, when using the same approach and formula to calculate the SMD for the two (sub-)groups in Kuehl et al. (2014a) for RECALL, we surprisingly obtained discrepant values as those reported by Xie and colleagues in Figure 2 (Xie et al. 2018: *SMD*_*system-paced*_ = −0.302; *SMD*_*self-paced*_ = −0.52; our values: *SMD*_*system-paced*_ = −0.489; *SMD*_*self-paced*_ = − 0.072). Given that both studies were relatively similar (e.g., both between-subjects design), we went back to rereading Xie’s and colleagues’ approach and both primary research studies to find an explanation for the discrepancy.

The values that Xie et al. (2018) reported could only be recreated when combining RECALL outcomes with PICTORIAL MATCHING outcomes for each (sub-)group in Kuehl et al. (2014a).^5^ Combining (sub-)groups in case of multiple reported outcome variables per single study, as well as selecting one outcome variable, is a *subjective* workflow decision. We could not find general information on rules for handling such cases with multiple outcomes in Xie’s and colleagues’ description “Calculation of Effect Sizes and Analysis.” Only general information was available, stating that for “multiple variates per study, we separately conducted analyses with regard to different dependent variables (i.e., recall test, transfer test, JOL, and learning time)” and “When a study reported multiple experiments or multiple conditions which were not related to the moderators, the data were merged to compute one pooled study-level effect size.” (see page 754 of Xie et al. 2018). In our opinion, the precise subjective workflow decisions regarding eligibility criteria for multiple outcomes (*not* conditions/(sub-)groups or experiments) and computational procedures are unclear and vague. Even if we interpret this to mean inclusion of a merger of multiple outcomes per study, there does not exist specific information for each experiment, leaving ambiguities overall concerning effect size calculations of primary studies.

Such ambiguities add up when considering the nested structure underlying a single reported effect size calculation of a primary study (e.g., multiple (sub-)groups and comparison opportunities per experiment; multiple outcomes per experiment; multiple experiments per study). This also includes different calculations for bias and interdependency correction. It is often unclear exactly which values were selected of which (sub-)groups. It is further unclear, if and how (sub-)groups were combined. The reasons for such subjective decisions (selection, merging, computation) are veiled, because often neither an explanation nor additional information nor the data or analysis specification underlying Xie’s et al.’s (2018) reported results was provided (cf. Lakens et al. 2016). This results in increasing inconsistencies between meta-analytical reported outcomes and reproduction attempts. In this respect, the examination of a primary study by Eitel et al. (2014) revealed further discrepancies.

The primary study by Eitel et al. (2014) comprised four experiments, each experiment with multiple outcome variables and three experiments with four experimental groups. Flexibility in effect size selection and calculation led Xie and colleagues to calculate an overall effect size of Eitel’s et al.’s Experiment 1 combined with a (sub-)group selection (text and picture conditions) of Experiment 2, combined with Experiment 3. Although we applied the same approach as the (in a “second” attempt) successful reproduction of the system-paced and self-paced effect sizes of Kuehl et al. (2014a), we could not reproduce the value of −0.476 that Xie et al. (2018) reported in their Figure 2 for RECALL (see file “Supplemental L1: RECALL SMD of Eitel and Kuehl (2016) Kuehl et al. (2014a) Eitel et al. (2014).xlsx”, https://osf.io/r6d5e/). Given the many options for selection, merging, and calculation, this could be dismissed as a consequence of ambiguity. Consequently, a reproduction failure could be seen simply as a failure to correctly interpret the general methodological information provided by the authors of the meta-analysis. However, it is not merely a matter of accurately interpreting the provided methodological information. In line with other meta-analytical reproduction attempts (e.g., Maassen et al. 2020), the main difficulties in reproduction of primary effect sizes are often due to ambiguities and insufficient information regarding precise effect size selection and calculation. This problem is more pronounced due to a lack of transparency regarding the underlying dataset and other resources, like analysis code, which would prove helpful in closing information gaps. The initially identified (minor) deviations and omission of information from the PRISMA guidelines (Moher et al. 2009, 2015) turned out to be more consequential, giving rise to inconsistencies and reproduction failures.

Such ambiguities also make classification more difficult, no matter if an unreproduced effect size is the result of a true error, stems from a mere inconsistency in the applied approach, or from different subjective choices. In this respect, we were puzzled when continuing to reproduce the reported effect sizes of Eitel et al. (2014) for the separately reported effect size of Experiment 2 (text only conditions) and Experiment 4. Applying the merging of RECALL outcomes and PICTORIAL RECALL outcomes for Experiment 4’s effect size yielded the same results that Xie et al. (2018) reported: a value of −0.108. Yet, for Experiment 2 (text only conditions), we obtained a value largely discrepant. The discrepancy could not be resolved in multiple attempts, so we do not know how Xie et al. (2018) obtained the effect size of −0.639, which they report in Figure 2 for RECALL. The results reported by Eitel et al. (2014) for the text-only condition do NOT suggest such a strong negative disfluency effect, neither for RECALL alone, nor for PICTORIAL RECALL alone, nor for a combination. The original values in the primary study for the text-only conditions in Eitel et al. (2014) indicate merely a small negative disfluency effect. Applying the approach of Xie et al. (2018), the effect size should be −0.14.

Besides the above presented failed reproductions and ambiguities regarding the strength of the recalculated effect sizes for RECALL and the abovementioned objective error in the coding of the mathematical sign (Rummer et al. 2016; Weissgerber and Reinhard 2017), we noticed further discrepancies throughout the reported results of Xie et al. (2018). In this respect, we discovered some minor mistakes with the classification of the moderating variables (see Supplemental L2-A, https://osf.io/49cuk/ and Supplemental L2-B, https://osf.io/5t9jn/). For example, authors (Weissgerber and Reinhard 2017) were categorized as having *no* test expectancy, but this is inaccurate: For the immediate posttest, the method section reports clearly that participants definitely expected a performance test. In contrast, the method section also reports that the delayed posttest was concealed from participants by means of a cover story. Weltman and Eakin (2014) were categorized as “no image” but their method section states: “Both words and figures were used to explain [...].” (page 158; Figure 2) with a depiction of the figure on the following page (page 159, Figure 3). Given that Weissgerber and Reinhard (2017) found no immediate disfluency effect but a delayed one, while Weltman and Eakin (2014) found a disfluency advantage, this misclassification is problematic and decreases the trustworthiness of the moderator analysis.

We further noticed that Xie’s and colleagues’ Figure 2 wrongly presents the learning times as “favors disfluent” when actually learning times in disfluent conditions are usually longer given greater reading difficulties; therefore, it should be labeled *learning times favor fluent fonts*, because shorter reading times equal greater time efficiency (and usually better outcomes). In contrast, Xie and colleagues correctly present Judgments of Learning (JOLs), which are usually lower for disfluent fonts and therefore favor fluent conditions, because lower ratings equal worse predictions about learning. Moreover, Xie’s and colleagues’ own definition states, “For Cohen’s *d*, the direction of the effect size was negative if recall, transfer, JOL, or learning time of the disfluent group was lower or shorter than that of the fluent group” (page 754 of Xie et al. 2018). This is confusing, and a broader readership unfamiliar with disfluency literature would be misinformed, misreading the direction of learning time effects.

While this can be seen as an excusable mistake and one minor inconsistency, inconsistencies are a frequent occurrence. These inconsistencies are symptomatic of a lack of transparency: The insufficient traceability makes it difficult to evaluate the soundness of certain subjective choices, especially since it is unclear whether Xie and colleagues obtained additional information from the authors of the primary studies that could justify otherwise questionable methodological choices. For example, Xie’s and colleagues’ Figure 2 reports TRANSFER performance of Eitel et al. (2014); however, TRANSFER is assessed in all four experiments of Eitel et al. (2014), but Experiment 1 to Experiment 3 only report means and standard errors *adjusted* for a covariate (spatial abilities), while Experiment 4 reports the *un*adjusted means and standard errors. Thus, a merging of all four experiments, as was seemingly done in Xie’s and colleagues’ Figure 2 for TRANSFER, is ill advised. Of course, it may be the case that Xie and colleagues obtained from the original authors the unadjusted scores without the covariate, making their computation correct. But the reader simply does not know. Of course, it could also be the case that the reported TRANSFER performance of Eitel’s and colleagues’ experiments (2014) by Xie and colleagues is the result of carefully selected (sub-)groups of the four experiments, making their computation justifiable. Likewise, the selected disfluency group 1 (sparing disfluency group 2 and disfluency group 3) of Seufert’s et al.’s (2017) study may be logical from Xie’s and colleagues’ perspective, but no necessary justification is provided for the reader; thus, Xie’s and colleagues’ methodological approach is *not* reproducible.

These further examples suggest that the meta-analysis was not thorough enough to establish sufficient confidence in the conclusions drawn. That a meta-analysis should be transparent and reproducible is not only important with regard to whether the overall results of the said meta-analysis are credible and based on accurate estimates; in essence, it is also crucial to gaining insight into the degree of idiosyncratic decisions of the underlying results. Reproducibility can thus be viewed from the perspective of researchers’ degree of freedom and flexibility in subjective choices. If ambiguities and lack of transparency exist, this cannot be achieved. We will not go into further detail here, and we do not wish to evoke the impression of discrediting the work of Xie and colleagues. We appreciate their efforts, but are obligated to point out these identified problems for the sake of self-correcting science. We see the present contribution as an opportunity to draw attention to some insufficient practices in the generation and review of meta-analyses to suggest stricter quality controls and to improve on quality standards. Our recommendations draw on the TOP-guidelines (Nosek et al. 2015) and guidelines by Lakens et al. (2016). We introduce Methods Reproducibility, which is organized in four levels (see our Table 1). In short, *Computational Reproducibility* (Level 1) and *Computational Verification* (Level 2) target the availability, comprehensibility, and accuracy of the underlying analysis code and reported results. *Analysis Reproducibility* (Level 3) and *Outcome Reproducibility* (Level 4) target reproducibility aspects that rest on researchers’ degrees of freedom regarding analytical choices and data coding choices. We describe disclosure preconditions related to the data, analysis code, and researchers’ workflows in our Table 1. The appendices and supplemental materials exemplify the approach regarding TRANSFER performance of Xie et al. (2018) for numerical validity (Level 2), the reproducibility of the analysis (Level 3), and the outcomes (Level 4).^6^

## Recommendations for Improving Meta-analytical Reproducibility

### Methods Reproducibility

Meta-analyses would benefit from a more rigorous adherence to open-science practices (e.g., open data, open code, and open access publications). The TOP-guidelines (Nosek et al. 2015; see https://osf.io/9f6gx/wiki/Guidelines/) provide general guidance, while the MARS- (APA 2008) and PRISMA guidelines (Moher et al. 2009) make more specific recommendations for meta-analyses. However, these guidelines are not always formulated specifically enough. Some aspects, like reproducibility, have become more important in the last few years and thus warrant further consideration. In this respect, two worthwhile goals are to increase the transparency and the reproducibility of meta-analytical results as a quality standard (cf. Lakens et al. 2016).

*Reproducibility* is broadly defined as “the ability of a researcher to duplicate the results of a prior study using the same materials and procedures as were used by the original investigator” (Bollen et al. 2015, page 3; Pellizzari et al. 2017, page 10). In practice, this implies as a minimum requirement that the data used are accessible (TOP-guidelines, Level 1; Nosek et al. 2015) and that (at least) the description of the coding and analytical approach in the meta-analysis report is sufficient for an independent researcher to recreate the analysis results within a reasonable margin of error. We think this minimum standard is outdated.

Further considerations of reproducibility offer a more fine-grained distinction of different reproducibility aspects (Goodman et al. 2016), of which we find Methods Reproducibility an important aspect for improving the quality of meta-analyses. *Methods Reproducibility* is “[...] the ability to implement, as exactly as possible, the experimental and computational procedures, with the same data and tools, to obtain the same results.” (Goodman et al. 2016, page 2). This definition has stricter implications for meta-analytical standards. We recommend following the Reproducibility Enhancement Principles (REP) in order to target the computational disclosure of data, code, and workflows (Stodden et al. 2016).

For Methods Reproducibility based on the REP-principles (Stodden et al. 2016), not only is data accessibility required, but so is access to the complete analysis code, including specifications about the software and version used to create the findings. This in turn requires knowledge about the workflow and subjective decisions on how raw data were selected, coded, and transformed. It requires additional insight into which intermediate results are further processed and in exactly what way; it also requires specific detail regarding which specified computational models the (intermediate) results are put in.

#### Computational Reproducibility—Level 1

REP-based (Stodden et al. 2016) Methods Reproducibility can be seen to establish reproducibility in a basic computational sense. We consider *Computational Reproducibility* as Level 1 to be most fundamental to Methods Reproducibility (see our Table 1). In line with others (e.g., Lakens et al. 2016; Stodden et al. 2016), we advocate for rigorous transparency about all the computational and analytical steps taken. This is necessary, since the aim is for an independent researcher to *exactly* recreate the reported results in the meta-analysis based on the data, workflows, and analysis code that the original authors provided (see TOP: Data Transparency, Level III; and TOP Analytic Methods (Code) Transparency, Level III; Nosek et al. 2015). In our opinion, it should thus be mandatory (and not voluntary) to publish the data set as a separate file alongside the manuscript (see TOP: Data Transparency, Level II; Nosek et al. 2015). Furthermore, it should be mandatory, prior to publication, to provide the complete analysis code used to create the findings (e.g., stored and published via a repository such as code ocean, Open Science Framework (OSF), etc.; see TOP Analytic Methods (Code) Transparency, Level II; Nosek et al. 2015; cf. Stodden et al. 2016).

Existing guidelines (e.g., Lakens et al. 2016; Maassen et al. 2020; Stodden et al. 2016) provide examples and specify the minimum analytical information required, for example, what needs to be included in the raw data file underlying the reported meta-analytical results: This starts with including all relevant texts from primary studies on which a coding decision or effect size computation is based and extends to statistical raw values (i.e., sample sizes for each condition, for each data point all test statistics and degrees of freedom, means, standard deviations, and correlations between dependent observations, etc.; see Lakens et al. 2016). We would like to highlight the valuable suggestions of Stodden et al. (2016) and Lakens et al. (2016) to “always include a link to data files that can be directly analyzed with statistical software, either by providing completely reproducible scripts containing both the data and the reported analyses in free software (e.g., R), or at the very minimum a spreadsheet that contains all meta-analytic data that can easily be analyzed in any statistical program” (Lakens et al. 2016); moreover, it is critical to outline details of the software and computational environment underlying the published findings in open trusted repositories, which should offer a permanent link and identifier in the publication for data, code, and digital information upon which the results depend (Stodden et al. 2016). In this respect, Lakens et al. (2016) outline the importance of “future-proofing meta-analyses” by enabling later re-analysis of the meta-analytical results as new statistical tools become available.

It is thus insufficient to merely provide the data and analysis code (TOP, Level II; Nosek et al. 2015) in the review process. However, the goal of Computational Reproducibility is that an independent researcher (i.e., reviewer) analytically repeats and evaluates the results reported in the manuscript and identifies potential issues and discrepancies in the produced outputs and reported results *prior* to publication (TOP, Level III; Nosek et al. 2015). The extent to which *exactly the same analyses results* can be obtained—given the same database and the same specified analytical tool and analytical approach *without the original authors’ aid* (self-sufficiency of provided information)—can be quantified as a percentage. This *reproduction percentage* reflects a comparison of all target values exactly reproduced in the recreated analysis output and all discrepancies obtained in the original values reported in the meta-analysis. A reproduction percentage less than 95% (e.g., allowing a few typos) requires explanation and correction from the original authors *prior* *to* *publication*.

For validation of Computational Reproducibility, the reviewer should verify that the data and code the authors provide is complete, usable, and understandable—that is, well-curated (see Hardwicke et al. 2018; Stodden et al. 2016). This includes specifications of the software and computational environment and this requires validating that a permanent link to the data, code, and tools are established. Verification is required so that after publication, independent researchers can completely (and also prospectively even later on) reproduce the reported meta-analytical results based on the provided sources.

#### Computational Verification—Level 2

Computational Reproducibility at Level 1 does not target validity checks, but merely ensures that the published results are numerically reproducible by recreating the reported analyses results based on the provided data (see also Hardwicke et al. 2018) and ensuring long-term accessibility. It is, however, highly desirable to check the data, the analysis code, and workflow for minor errors or (procedural) mistakes and any uncertainties to ensure accurate data and analysis code. Therefore, Level 2 of Methods Reproducibility should target the *Computational Verification*, that is the accuracy of the general analysis approach, and specific analysis steps taken (within the analytical logic and subjective decisions of the original authors of the meta-analysis, not what oneself considers appropriate). Although evaluating the general analysis approach is traditionally part of the review process, our Level 2 check (Computational Verification) suggests a much more detail-focused, in-depth evaluation.

We not only advocate for greater transparency of the analytical approach and analytical tools, but also for transparency in all subjective decisions made in creating the database (TOP Design and Analysis Transparency (Level II) refers to this as “other documentation of the research process”, Nosek et al. 2015; https://osf.io/9f6gx/wiki/Guidelines/). This requires detailed information on workflow steps, including what subjective decisions have been made, such as coding or (sub-)group merging, for example, as well as verification of those steps and decisions (TOP, Level III; Nosek et al. 2015; cf. Lakens et al. 2016; Stodden et al. 2016).

For example, the data file must include the original values, extracted from the primary studies or otherwise obtained, which were used to compute the effect sizes. Not all data come directly from primary studies; occasionally information and data must be obtained directly from the authors. Thus, a thorough list of additional data and information obtained but not included in primary studies is advisable (cf. Lakens et al. 2016; Stodden et al. 2016). It is highly recommended to publish a coding booklet with comments logging subjective decisions that had to be made throughout the data coding phase. Recording the chosen forks or at least alerting readers to the relevant knots in the “garden of forking paths” is important, because even data selection and coding decisions can be highly contingent on the data (Gelman and Loken 2013).

This record of subjective coding decisions is relevant in that it allows for a check of the consistent use of specifications within and between primary studies. In primary studies, it is often more or less obvious at first glance which specifications serve specific research questions, even before researchers have any idea of what (further) decisions are to be made during the coding process. Sometimes those decisions are arbitrary. Therefore, even though someone tries to be objective, unintentional biases cannot be avoided. Forcing coders to write down their workflow decisions reduces intentional and unintentional errors, at least in the ongoing coding process. The advantage lies in being able to check the consistency of decision making. Logging workflow decisions is thus not only crucial for the reviewers, but also for the researchers in the first place.

A well-curated codebook and analysis code help detect minor mistakes or inconsistencies in the analysis code (e.g., unintentional inclusion of the untransformed variable or wrong variable in the effect size computations). The formulas used for computation should be included by default in the appendix or supplemental materials as a formula collection. For example, it needs to be clear which calculations are used for effect sizes based on different designs, such as within-/between-/mixed-subjects designs or whether (and which) variable transformations were used (cf. Lakens et al. 2016). This is especially important because the use of meta-analytical software tools, such as Comprehensive Meta-Analysis Software (CMA), can decrease the workload of conducting a meta-analysis. This may of course pose the disadvantage of the software automatizing certain steps, such as effect size calculation. Such automatization then requires authors to manually request the program to show what it computed, how it did so, and based on what formula. For example, if requested, CMA does an automatic merging of within-study (sub-)groups. When using such tools, emphasis should be placed in particular on transparency and traceability (disclosure of automated decisions).^7^ Again, the suggestions to create completely reproducible scripts including the data in open-source software or preserving the computational steps in open-source repositories are recommended (Lakens et al. 2016; Stodden et al. 2016).

As such, for Computational Verification, the original authors not only provide the *original data* (including raw data used to estimate effect sizes and further computed variables), the *original analysis code* (understandable), and a *description of the applied analysis tools* (software and version, etc.). They also submit a *codebook* (well-curated), the *original workflow* (including all processing steps such as coding or (sub-)group merging) complemented by a *log of subjective decisions* (e.g., study, (sub-)group, effect size selection, and coding decisions), *additionally obtained information* (e.g., from authors of primary studies), and the *computational formulas* (especially for dealing with different design variants).

Adopting ideas of Lakens and colleagues (2016), the task of the reviewer for Computational Verification (Level 2) is to check four main sources of error: (1) *Erroneous calculation of effect sizes*, for example, correct coding of study characteristics and accurate extraction of each primary effect size or utilized parameters, including consistent coding throughout. We extend Lakens et al. (2016) and add that checking erroneous calculations of effect sizes should explicitly entail verifying whether independencies underlying merged effect sizes are (correctly) considered and biases are appropriately adjusted computationally. (2) *Inconsistent calculation of effect sizes*, for example, consistent parameter choice such as not switching between main effect versus simple effects of interactions. We like to add that this includes in particular checks of consistent choices to combine outcomes, (sub-)groups and experimental conditions, and merging of effects across experiments and primary studies. (3) *Incorrect inclusion of effect sizes*, for example, verifying the sensibility of (often subjective) in-/exclusion criteria and consistent implementation throughout. (4) *Incorrect calculation of meta-analytic effect sizes*, for example, whether assumptions and the chosen models are appropriate, such as fixed or random-effects model or meta-regression, and whether the computations were accurately implemented. The validation of these four error sources includes the review of the subjective workflow choices and their consistent adoption. If a preregistered meta-analysis protocol exists, Level 2 (Computational Verification) further requires verification that the planned data coding and analysis approach are consistent with the data actually provided and with the reported analysis.

If a database or analysis approach rests on greater degrees of freedom (with many subjective decisions or data-contingent choices that had to be made based on the primary studies), more advanced levels of Methods Reproducibility than Level 1 and Level 2 are of great interest. Both Analysis Reproducibility (Level 3) and Outcome Reproducibility (Level 4) offer the evaluation of reproducibility aspects that rest on researchers’ degrees of freedom regarding data set creation and analytical choices (see Table 1). Level 3 and Level 4 address the issue of how others (including reviewers) can examine how sensitive the meta-analytical results are to subjective choices.

#### Analysis Reproducibility—Level 3

*Analysis Reproducibility* (Level 3) relates to the fact that there is no single correct way to analyze data. Instead, multiple models can be rightfully fitted. Moreover, different effect size metrics can be rightfully used and different reasons for in-/exclusion of data subsets and control variables can be rightfully devised (cf. Lakens et al. 2016). Some analysis approaches and fitted models may be highly similar to the analysis approach and to models utilized by the original authors of the meta-analysis; however, multiple different but equally plausible and defensible analysis approaches exist as well. Analytical choices affect results (Silberzahn et al. 2018) and thus reproducibility. Yet, running all plausible models via specification curve (Simonsohn et al. 2015) might prove unfeasible for meta-analyses to capture results and effect size variation due to flexibility in data analysis. Doing so for the multitude of outcome variables reported in some meta-analyses is rather unrealistic. Instead, Voracek et al. (2019) suggest a multiverse-analysis approach for meta-analyses identifying theoretically guided eligible specifications that are put into a specification curve.^8^ The authors provide a complete worked example and step-by-step guide for meta-analyses. We highly recommend adopting their proposed multiverse and specification-curve approach. The approach allows for an excellent summary of effect size variations depending on different analysis approaches and analysis specifications. The specification-curve then puts into perspective the analysis approach chosen by the original authors and the obtained effect sizes: A comparison of the specification-curve results and the original results illuminates the “strangeness” of the attained effect sizes based on the original author’s analysis approach. The stranger—that is, the more the original authors’ effect size(s) deviate from the effect size distribution of all the alternative analytical models, the less robust and credible are the obtained meta-analytical effect size(s) of the original meta-analysis.

If specification-curve (Voracek et al. 2019) seems unfeasible, we at least suggest applying one’s own analytical approach and analytical tool based on a sub-sample of the provided data by the original authors and one randomly or strategically chosen dependent variable of the originally reported results. For example, in Xie’s and colleagues’ meta-analysis, this could be only the data pertaining to the studies that gave rise to the reported TRANSFER performance in their Figure 2. In this respect, we think it is worthwhile to follow a two-fold strategy. The first step is to take a similar analysis approach by fitting *similar* plausible models as in the original report (*not the same* model, as this concerns Level 1). The goal is to check whether *results similar* to those of the original authors can be reproduced based on *similar analyses and models.* The second step is to identify multiple different but plausible and defensible analysis approaches; the goal is to check whether *results similar* to those of the original authors can be reproduced based on the most plausible set of *DISsimilar analyses and models*.

An instability in the meta-analytical results based on the variability in analytical choices is highly valuable information. Documentation of the analysis code and adopted computational formulas is particularly important. Aligning one’s work to support Level 3 reproducibility is much more complex than establishing Computational Reproducibility at Level 1 or Computational Verification at Level 2. It is worthwhile, though, because it can increase trust in the (to-be-) published results, and it allows for methodological validation.

Establishing Analysis Reproducibility (Level 3) relies on the same information sources as Computational Verification (Level 2; data, code, workflows including subjective decision logs etc.) but offers more in-depth exploration and evaluation of potential discrepancies. In this respect, checks of Analysis Reproducibility should be planned beforehand: It is ideal to deposit and agree on a plan (when conducted as part of the review process) or preregister a *reproducibility protocol* (when conducted after publication) on what alternative analytical approaches are to be conducted. Importantly, criteria for quantifying and evaluating the degree of discrepancy between original reported meta-analytic results and alternative analysis approaches need to be established beforehand (e.g., focus on overall effect size estimate or confidence interval, and quantification metric, such as classification of discrepancy in correlation or Cohen’s d; see Maassen et al. 2020). The reason is simply that analytical approaches and chosen evaluation criteria are not inherently objective, but contingent on subjective views. Evaluating the discrepancy between original meta-analytical results and the results based on alternative subjective approaches should limit reviewers’ or reproducers’ degrees of freedom in evaluations to avoid a bias in the appraisal in favor or against the original reported results.

#### Outcome Reproducibility—Level 4

Level 4 (Outcome Reproducibility) is similar to Level 3 (Analysis Reproducibility) in conducting specification curve analysis, or applying one’s own analytical tools and approach. However, Level 4 is extended by one further requirement related to the database.^9^ Level 4 *forbids the use of the provided data by the authors* and instead requires researchers to *(re-)create a (small) portion of the database*: The subset of the studies relevant for the results of one or more systematically or randomly chosen output variable(s), reported in the original results, must be coded again. We think that this is relevant because the PRISMA guidelines (Moher et al. 2009: item #9; Moher et al. 2015: item #11b) propose a general and hardly comprehensible solution. For example, the original authors should (merely) describe that contradictory codings were resolved by discussion (and not even which criteria were applied to solve coding discrepancies). The (re-)creation of a portion of the database does, however, pose multiple benefits. It functions to identify potential coding mistakes and inappropriate choices, for instance with (sub-)group selection. Furthermore, consistent use of specification within and between primary studies can be checked. The most important function is to include the coding when deliberately different data-contingent coding decisions are made. This is paramount, especially when a paper reports many potentially relevant effect sizes in multiple studies and selection choices must be made, or when debatable criteria for study eligibility exist. This (re-)creation of the database allows to compute alternative results based on different subjective decisions. It is possible to inspect whether and how much the recalculated results deviate (when applying the same analytical tools and approach by the original authors as used in Level 1 for Computational Reproducibility, or while applying different analytical choices as the original authors as in Level 3; see meta-analytical specification curve; Voracek et al. 2019). In other words, Outcome Reproducibility focuses on (a) outcome variability in the results based on *subjective decisions related to the creation of the database* (when using the original analysis approach of the original authors; Level 1 Computational Reproducibility) and (b) outcome variability in the results based *on subjective analytical choices* (that is, Analysis Reproducibility of Level 3).

Establishing Outcome Reproducibility (Level 4) requires the same disclosed information sources as Computational Verification (Level 2; the availability of the data, the analysis code, the detailed workflows, the log with additionally obtained data or information, the code book, and the record of subjective coding decisions in an accurate and understandable manner).

Similar to Level 3 checks on Analysis Reproducibility, on the side of the reviewer or independent researcher conducting Level 4 checks on Outcome Reproducibility, these checks require depositing beforehand a reproducibility plan or preregistered reproducibility protocol including evaluation criteria and metrics to avoid bias.

## Implications and Desirable Changes to the Generation and Review of Meta-analyses

Current reporting of meta-analyses often does not meet the standards of transparency and reproducibility. In this sense, one of our core requirements is to place more emphasis on authors, reviewers, and journals in following more stringently open-science practices: they promote transparency and reproducibility and can thus contribute to quality gains. We acknowledge that more rigorous standards for meta-analyses place greater work demands on the authors (see our Table 1, second row titled “original authors”). However, our proposal has implications for the review process of meta-analyses, too. Journals should select at least one reviewer with meta-analytical expertise. We acknowledge that reviewing a meta-analysis is no easy task, requiring specific and thorough methodological knowledge. However, surface-level checks by non-experts, although familiar with the respective research area, are insufficient, as this example of Xie and colleagues demonstrates. Therefore, the reviewer teams selected by the editor should include both an expert familiar with the research area and topic and a proven meta-analytical expert.

Moreover, the journal policy should *always* require publishing the curated data file and analysis code. Transparency as a fundamental requirement is mandatory. Multiple journals have successfully introduced open data badges (e.g., Kidwell et al. 2016), and introducing award code badges to authors may be a promising tool. In line with suggestions of the TOP-guidelines (Nosek et al. 2015) recommending journals to declare which TOP-Levels are fulfilled, we suggest that journals award *Reproducibility badges*, specifying at which Level (1 to 4) reproducibility was established beforehand. Similar to disclosure of conflicts of interest, journals could request authors to explicitely declare who is responsible for ensuring which reproducibility level.

In our view, a minimum of one qualified person, ideally a journal associate or alternatively a reviewer, independent researcher, or designated co-author, is to check methodological reproducibility at Level 1 (Computational Reproducibility) and at Level 2 (Computational Verification; see Table 1). Crucially, at least one additional person (e.g., qualified journal associate, reviewer, independent researcher, or designated co-author) should be solely responsible for verifying the adequacy of the used analytical approach and the stability of the analysis results at Level 3 (Analysis Reproducibility). We recommend that one person establishes Outcome Reproducibility at Level 4 (Outcome Reproducibility). *Whether or not Methods Reproducibility is established and at which level and by whom (journal associate, reviewer, or designated co-author) should be explicitly and visibly denoted in the manuscript prior to publication.*

We are aware that establishing Methods Reproducibility at any level requires increased workloads. We think that establishing Computational Reproducibility (Level 1) and Computational Verification (Level 2) is mandatory *prior* to publication. However, due to the increased workload of Analysis Reproducibility (Level 3) and Outcome Reproducibility (Level 4), a more realistic scenario would be that journals invite independent researchers to publish a commentary alongside the meta-analysis (e.g., similar to the format Open Peer Commentary in the journal *Behavioral and Brain Sciences*, where commentaries related to a target article are published together with the target article; www.cambridge.org/core/journals/behavioral-and-brain-sciences). This commentary should address reproducibility aspects and include the meta-analytical results of Level 3 and Level 4 checks (Analysis and Outcome Reproducibility). This commentary option may be a desirable incentive for independent researchers (or reviewers) to conduct in-depth reproducibility checks because of providing a publication opportunity. Although we advocate creating Methods Reproducibility prior to publication, we acknowledge that this may seem a radical and resource-intensive change to journal workflows; still, in our opinion, increasing trust and confidence in research findings is worth the effort.

In any case, we believe it unwise to leave the quality checking to the reader, who in most cases will not be an expert in meta-analysis or on the topic at hand. Systematic errors are then unlikely to be detected.^10^ It is also undesirable that journals leave the responsibility to reviewers alone, especially without providing proper acknowledgement or compensation. As an ideal long-term development, we hope that a growing demand for transparency and reproducibility from researchers motivates journals to employ data scientists whose task is to aid reproducibility and to facilitate the methodological quality of (meta-analytical) manuscripts prior to publication. This may seem an unrealistic suggestion for manuscripts in general. In contrast, for meta-analyses alone, the high impact journals could use this as an opportunity to distinguish themselves by guaranteeing meta-analytic quality. Employing data scientists to improve reproducibility and methodological quality clearly positions and strongly signals a journal’s support of adhering to the highest standards of scientific rigor.

At least for now, we hope that more co-authors take responsibility to carefully check reproducibility aspects of their manuscripts prior to submission. We strongly suggest that journals require authors to explicitly declare whether and to which standard (e.g., TOP-Levels, Nosek et al. 2015; or see our Table 1) authors support reproducibility. Again, awarding a badge to authors for quality control may be an option. We would like to stress that independent researchers (or reviewers) who invest a significant amount of time in evaluating Methods Reproducibility deserve some recognition, depending on the level of effort. At the very least, they deserve an explicit honorary mention or, in some cases, co-authorship (see CRediT; www.casrai.org/credit.html; e.g., Allen et al. 2019) or the chance to publish an invited commentary on the meta-analysis’s reproducibility alongside the meta-analysis (see above). We would like to note that these suggested changes to the review process are *NOT to be implemented as a further criterion for rejecting manuscripts but as quality management prior to publication.*

In the context of current self-renewal and self-improvement efforts in science, we like to stress the need to apply more rigorous standards for meta-analyses. We appeal to the journals and editors, whom we see as having a responsibility to implement better quality control and improve the review process. Transparency and reproducibility must both be demanded and supplied by authors and journals alike.

### Are the Meta-analytical Results of Xie et al. (2018) Null and Void?

To reflect on this question, we first would like to emphasize that the goal of the present paper was to tackle current problems in the transparency and reproducibility of meta-analyses. Our goal was by no means to discredit the work of Xie et al. (2018) or to search for as many mistakes we could find. We have therefore placed the focus of this paper on introducing recommendations for improving Methods Reproducibility of meta-analyses by presenting a systematic approach that targets Computational Reproducibility (Level 1), Computational Verification (Level 2), Analysis Reproducibility (Level 3), and Outcome Reproducibility (Level 4). The meta-analysis of Xie and colleagues served as a hook and background example to illustrate the approach. Therefore, we did *not* centrally organize the paper in a way that adopts the proposed checks and then points out the results and problems in the main text of the present paper. We consciously chose the main text to be about introducing Methods Reproducibility and about having detailed results of the checks of the work of Xie et al. (2018) as supplementary documents on our osf page (https://osf.io/nvhqz/).

Again, since our goal was *not* to uncover problems in the meta-analysis of Xie et al. (2018), we tried to be objective, transparent, and benevolent when applying the proposed checks of Methods Reproducibility. In this respect, one of the reasons we chose to apply our checks of Level 3 (Analysis Reproducibility) and Level 4 (Outcome Reproducibility) on TRANSFER performance was that we expected many fewer inconsistencies, errors, and deviations for TRANSFER as outcome variable than when checking RECALL. The reason is simply that TRANSFER performance was based on a much smaller number of studies than RECALL and thus inherently entails much less flexibility of subjective choices related to the creation of the database and related to flexibility in subjective analytical choices. Hence, we expected the results of the reproducibility checks of TRANSFER performance to be favorable and to *not* produce results largely different from the meta-analytical findings of Xie et al. (2018).

After having conducted the checks on Analysis Reproducibility (Level 3; see Appendix L3: Similar and DISsimilar analyses approach, https://osf.io/zdf8v/) and Outcome Reproducibility (Level 4; see Appendix L4: Similar and DISsimilar analyses approach, Table 1, https://osf.io/jwng6/), our initial thinking is partially correct and partially incorrect—correct because we found just one coding error^11^ for TRANSFER performance as outcome variable, namely in the coding of Eitel et al. (2014), albeit producing a difference in SMD of 0.18 when corrected (see Appendix L2: Check of dataset, effect sizes, and workflow, https://osf.io/7gj23/; but also https://osf.io/5t9jn/ and https://osf.io/49cuk/). Fortunately, the coding error produced only a small overall deviation of *Δ* = 0.034 (Xie et al. 2018: pooled effect size of +0.026; corrected pooled effect size: −0.008). Thus, it did not change the *overall* null-effect of disfluency on TRANSFER performance when applying a *similar analysis* approach as Xie et al. and *making the same subjective decisions regarding the creation of the database* in Level 3 checks of Analytical Reproducibility. However, when applying a *DISsimilar analysis*—one that we think is theoretically and methodically more advantageous—the deviation becomes more pronounced (*Δ* = 0.089) but it is still small (from a clear-cut null-effect of disfluency of −0.008 to a small positive pooled effect of *+*0.081)*.*

While checks of Analysis Reproducibility are based on the “original” dataset, checks of Outcome Reproducibility require the recreation (of parts) of the database by the independent coding of primary studies. Level 3 checks of similar and DISsimilar analysis approaches are then repeated in this new database. When taking into account the flexibility in choices related to the creation of the database –—hence creating our *own database*—and choosing a *similar analysis* as Xie et al. (2018), we found a small deviation of *Δ* = 0.023 (*not* affecting the overall null-finding). Adopting a *DISsimilar analysis* approach, we found a small deviation of *Δ* = 0.069 to indicate a small positive pooled effect of disfluency on TRANSFER (+0.061), which is likely not significantly different from zero. The combined effect of flexibility in analytical choices and flexibility in the creation of the database amounted to a maximum difference of *Δ* = 0.112 (minimum obtained pooled effect size = −0.031; maximum obtained pooled effect size = +0.089). Importantly, this applies *only* to the possibilities we tested using the specific combinations of subjective choices we outlined (see “Supplemental L3-A: Our subjective decision tree (multiverse pool)”, https://osf.io/2k86t/; and Supplemental L3-B: Our workflow (subjective decisions), https://osf.io/eyvdp/). However, we used the most likely and reasonable choices for database creation (for example, choices preserving information and no extreme choices that are more focused on certain (sub-)groups, such as only disfluency manipulations being very similar or being the same as the font manipulations of the paper by Diemand-Yauman and colleagues 2011).

We also utilized highly likely analytical choices and tested our initial claim that combining covariate-corrected results and uncorrected results reported in primary studies may be less than ideal for effect size calculation by means of our moderator analysis (see https://osf.io/zdf8v/: page 3; and https://osf.io/jwng6/: page 3). The results of the moderator analysis indeed show greater heterogeneity of covariate-corrected results in primary studies and on average a more negative effect as uncorrected results. As such, the raw values of primary studies should be obtained, given that authors of primary studies included different covariates for correction across studies, for instance, need for cognition (Kuehl et al. 2014a), reading comprehension (Eitel and Kuehl 2016), and spatial abilities (Eitel et al. 2014). Therefore, what has been said in terms of low to moderate variability as a result of Analysis Reproducibility and Outcome Reproducibility checks is to be relativized considering the unknown change of results when covariate-uncorrected results would be obtained.

Finally, we need to consider what we did *not* learn from Level 3 and Level 4 checks about *Analytical Reproducibility and Outcome Reproducibility* in Xie et al. (2018) for TRANSFER performance. Since we chose TRANSFER performance as an outcome variable and *not* RECALL, which consists of a much larger set of primary studies and many more options, as well as allowing for much more flexibility of subjective decisions (e.g., as fundamental as *not* to combine disfluency manipulations of size and font in contrast to Xie et al. 2018), we cannot say anything reliable about reproducibility with respect to the results for other outcomes in Xie et al. (2018). Thus, we cannot draw conclusions about the generalizability, especially regarding changes in the results of the moderator analysis for RECALL. Given the objective errors regarding algebraic sign, coding decisions, and so forth—and as mentioned in the introduction— we remain more skeptical here.

All in all, at least with respect to our conducted reproducibility checks, the results of Xie 's et al.’s (2018) appear robust. Given that the results do not deviate much, do we really need all of this checking? Is it worth the trouble? As Gettier (1963) pointed out, obtaining a correct result based on luck does *not* constitute knowledge. Although we believe that the null-effect Xie et al. (2018) found could be the “correct” summary across all disfluency studies, we suspect this to be the case due to favorable coincidences coming together (for example, switching the algebraic sign of a study showing a positive effect toward a negative effect (Weissgerber and Reinhard 2017) and switching the algebraic sign of a study showing a negative effect toward a positive effect (e.g., Eitel et al. 2014: Experiment 3; Rummer et al. 2016, Experiment 2). While the deviations are not in particular consequential for the overall classification of the pooled effect into such broad categories as “null,” “negative,” or “positive,” when it comes to *precision* regarding the estimate of the effect sizes, deviations are problematic (especially for results from moderator analysis and for potential real-world interventions).

Thus, we still must conclude that the identified problems in the meta-analysis raise *doubts* about the validity of the reported results and the conclusions drawn. Hence, it is still unknown whether the findings of Xie et al. (2018) are null and void. We recommend that the transparency and reproducibility underlying the meta-analysis of Xie et al. (2018)—and many other meta-analyses—can and should be improved. In our opinion, the credibility and trust in scientific findings are goods that must be actively created and maintained, where reproducibility alongside replicability act as important corner stones that mark good scientific practice (Popper 1959).

## Limitations and Future Directions

Although we presented a more grounded approach (see Table 1) to advance efforts regarding meta-analytical transparency and reproducibility, here like to briefly summarize the limitations in our work and the application of our framework to Xie et al. (2018).

Scrutinizing Computational Reproducibility (Level 1) checks whether the results are numerically exactly the same when independently reproduced. Since Xie and colleagues provided no raw data underlying effect size calculation or code files, we could not run Level 1 checks of Computational Reproducibility as outlined. Instead we adopted a variant. First, we compiled a document and applied PRISMA-checks (see Appendix L1, https://osf.io/5v8m2/) to get an overview of the completeness, usability, and understandability of the provided information in the meta-analysis. We focused in particular on information about the effect size selection of primary studies and effect size computation to reconstruct Xie’s et al.’s (2018) approach. Second, to exactly numerically reproduce (at least some) effect sizes that Xie et al. (2018) reported their in Figure 1, we made a strategic decision to a) check RECALL papers and b) to focus on papers authored by Eitel and/or Kuehl given their active contribution to the disfluency literature (e.g., Eitel et al. 2014; Kuehl et al. 2014a, b; Eitel and Kuehl 2016), trying multiple different ways of selection and computation (see Supplemental L1: RECALL SMD of Eitel and Kuehl (2016) Kuehl et al. (2014a) Eitel et al. (2014), https://osf.io/r6d5e/). Both were subjective decisions. Thus, Level 1 checks were not possible—and our adopted variant represents a first selective look but not a(*n* exact) recalculation of all reported effect sizes in Figure 1 of Xie et al. (2018). Level 1 checks rely on the availability of data and computational code. In the case that Xie et al. (2018) make their data and code available in the future, other researchers may check the numerical accuracy of their entire results (Level 1).

Since Level 2 checks build on Level 1 checks, we could not conduct Level 2 checks on Computational Verification as outlined, but we adopted an indirect variant of Level 2 checks (see Appendix L2, https://osf.io/7gj23/; and also https://osf.io/49cuk/ and https://osf.io/5t9jn/). For a more complete and thorough check, we decided to focus on all papers under TRANSFER. Since we were not provided with the original database underlying the work of Xie et al. (2018), we had to rebuild the “original” database. We recreated the “original” database and workflow based on the effect sizes reported in Figure 2 for TRANSFER by trying to reproduce the analytical choices that Xie et al. (2018) may have made to obtain the effect sizes in their Figure 2. Thus, instead of being directly able to check the dataset, analysis code, and workflow for errors, we had to reconstruct Xie’s et al.’s (2018) most likely approach, and then check for errors and workflow inconsistencies as outlined by Level 2. Choosing TRANSFER and using the free trial version of CMA to recalculate the effect sizes and R’s metafor package were subjective choices. Thus, Level 2 checks are an approximation; our adopted variant was carried out on the recreated “original” data and workflow of Xie et al. (2018) as closely as possible as outlined in the present paper. Still, we could not recreate and check all “original” data and workflow decisions underlying all reported numbers by Xie et al. (2018) in their Figure 1 and 2 and Tables 1 to 4. Other researchers may check our documents and verify our conclusions then extend their examination beyond TRANSFER performance (all files and datasets can be found here: https://osf.io/nvhqz/).

The goal of Level 3 checks (Analytical Reproducibility) was to see whether the conducted analyses were robust and reproducible in light of researchers’ flexibility in analytical choices. To this end, we examined the variability in the pooled effect size for TRANSFER performance reported by Xie et al. (2018). We used the recreated “original” dataset (copying any values from Xie that we could not exactly reproduce). We applied our own similar analytical approach based on our reconstruction of Xie’s and colleagues’ approach, and we illustrated how to apply one’s own DISsimilar analytical approach. We chose several—but not all possible—DISsimilar analysis approaches as a combination of various computational options and subjective workflow choices performed on the “original” dataset. We chose approaches which were plausible and reasonably different but very likely to be implemented by other researchers. Thus, future work could choose RECALL instead of TRANSFER and explore other DISsimilar analytical choices we did not apply. Furthermore, based on Voracek’s et al.’s (2019) worked example, future work could adopt a multiverse and specification-curve approach to Xie’s et al.’s meta-analysis.

In Level 4 checks on Outcome Reproducibility, we created our own database and performed similar and DISsimilar analytical choices. Again, for dataset creation and analytical choices, we chose several but not all possible DISsimilar analysis approaches as a combination of various computational options and subjective workflow choices performed on the “original” dataset. Since we chose TRANSFER performance as outcome variable and not RECALL—with a much larger set of original studies and many more options and flexibility of subjective decisions—we cannot say anything reliable with respect to results for other dependent variables, such as recall or learning times in Xie et al. (2018). In other words, we cannot make reliable claims about the generalizability to other outcomes in Xie’s and colleagues’ work or about the variability based on other possible options for subjective choices not covered in our workflow; for example, our findings regarding the obtained variation apply only to reasonable, and to the most likely, choices preserving information (e.g., not to choices that are more focused on certain (sub-)groups). Future work could thus adopt more “unusual” choices for data set creation and for the DISsimilar analytical choices. Moreover, we want to encourage future researchers to apply a multiverse and specification-curve approach to Xie’s et al.’s meta-analysis (cf. Voracek et al. 2019).

One general shortcoming in our work is that we did not deposit beforehand a *specific* reproducibility plan or preregistered protocol including evaluation criteria and metrics, although our framework can be understood as a general plan.

We tried to get in contact via email with Xie and colleagues, but maybe due to the outbreak of the Covid-19 pandemic, we did not receive a response. This meant that we could not directly communicate with Xie and colleagues to resolve potential misunderstandings or unclarities in the description in their methods and results section. Taken together, we think one way that future work could build on our work would be to conceive a Registered Replication Report of Xie’s et al.’s meta-analysis (2018) then have it reviewed by a suitable journal and coordinated with Xie and colleagues. The published supplemental documents (e.g., data and code) could be used in the future for an updated or new meta-analysis on disfluency (cf. Lakens et al. 2016: future-proofing of meta-analysis).

We think it would be beneficial, if future meta-analyses on the disfluency effect not only attend to heterogeneity within primary studies and calculated effect sizes (moderators), but also comment on a potential heterogeneity across (future) meta-analyses and to biases (e.g., selections and subjective choices). From an applied perspective, the disfluency effect (better learning after harder-to-read texts) is intriguing, because it could be easily and cheaply applied in real-world contexts—if it were reliably established whether disfluency works in general and/or under which circumstances. This, however, requires careful methodological conduct and attention to heterogeneity to allow any comment on the Evidence Readiness Levels (ERL; IJzerman et al. 2020). The Evidence Readiness Level framework is a scheme to assess the quality of scientific evidence prior to real-world application and policy. For example, ERL Level 3 requires carrying out of systematic reviews and meta-syntheses to select evidence that could potentially be applied. Transparent and reproducible meta-analytical findings are paramount in establishing reliable insights as a base for further efforts on which scalable and generalizable interventions are scaffolded (Evidence Readiness Level 7). For to-be-applied findings—like the disfluency effect—credible science should be able to establish robustly and transparently whether evidence suffices for beneficial real-world impact.

## Footnotes

1 Note: The focus is on presenting a systematic framework not on introducing new analysis tools.

2 Although Xie and colleagues listed all outcome variables of interest (RECALL performance, TRANSFER performance, Judgments of Learning - abbr. JOL, learning times), the outcome variables were not defined (e.g., what outcome measure constitutes TRANSFER; PRISMA-P, 2015: #13, Moher et al. 2015).

3 For our calculations of the SMD, please see file “Supplemental L1: RECALL SMD of Eitel and Kuehl (2016) Kuehl et al. (2014a) Eitel et al. (2014).xlsx”. For broad accessibility, our recalculations are in excel calculation format (see https://osf.io/r6d5e/).

4 For example, Cohen’s d as effect size; calculated as mean score difference between a disfluent and a fluent group; negative sign, if outcome of the disfluent group was lower or shorter than that of the fluent group.

5 Note: PICTORIAL MATCHING was assessed as outcome variable in Kuehl et al. (2014a), but not in Eitel et al. (2014). This seems to “explain” the discrepancy, but this is insider knowledge that requires explicit mentioning in subjective workflow decisions for reproducibility, and apparently combining PICTORIAL MATCHING and RECALL was *not* consistently implemented (see https://osf.io/r6d5e/).

6 Level 1 checks on Computational Reproducibility could not be demonstrated because we did not have the necessary information of Xie et al. (2018), e.g., no original data set and analysis code or complete workflow.

7 Note: Xie and colleagues used CMA, and some of their lack of transparency might simply stem from their particular usage of the CMA tool.

8 As specification-curve for meta-analysis has just very recently devised (Voracek et al. 2019), almost all meta-analysis will not have this approach included. If in future meta-analysis, specification-curves become more common practice, the accuracy of the specification-curve computations and outcomes should be verified.

9 Note: Goodman’s and colleagues’ (2016) definition of Methods Reproducibility emphasizes precision regarding the implementation of the computational procedures “as exactly as possible, with the same data and tools, to obtain the same results.” (Goodman et al. 2016, page 2). This is reflected in our Level 1 conceptualization of Methods Reproducibility as Computational Reproducibility. Our conception of Level 3’s Analytical Reproducibility builds on but necessarily removes this precision constraint by considering researcher’s flexibility in analytical choices (and tools). Likewise, our conceptualization of Level 4 further removes the constraint of using *exactly* the same data source. The logic of the four levels is to move from reliability to include validity aspects.

10 Anecdotally, the inconsistencies discovered in Xie’s and colleagues’ meta-analysis were driven mainly by the fact that we are ourselves authors of primary studies, experts on the topic who, by chance, happen to work closely with other colleagues knowledgeable in meta-analysis.

11 We found further coding errors for TRANSFER related to the *moderator* variables (listed in Table 1 of Xie et al. 2018; see our documents “Supplemental L2-A: OVERVIEW check coding decisions Table 1 of Xie (TRANSFER).xlsx” and “Supplemental L2-B: INFO check coding decisions Table 1 of Xie (TRANSFER).docx” (https://osf.io/49cuk/ and https://osf.io/5t9jn/). These coding errors were *inconsequential*, because Xie et al. (2018) did *not* conduct moderator analysis for TRANSFER, likely due to the limited number of studies.
